# An Enhanced Tree-Seed Algorithm for Function Optimization and Production Optimization

**DOI:** 10.3390/biomimetics9060334

**Published:** 2024-05-31

**Authors:** Qingan Zhou, Rong Dai, Guoxiao Zhou, Shenghui Ma, Shunshe Luo

**Affiliations:** 1 School of Geoscience, Yangtze University, Wuhan 430100, China; qganzhou@163.com; 2 School of Physics and Optoelectronic Engineering, Yangtze University, Jingzhou 434023, China; 3 PetroChina Changqing Oil Field Company, Research Institute of Exploration and Development, Xi’an 710018, China; goxiaozhou@163.com (G.Z.); senghuima@163.com (S.M.); 4 Cooperative lnnovation Center of Unconventional Oil and Gas (Yangtze University), Wuhan 430100, China; luoshunshe_ytu@163.com

**Keywords:** evolutionary algorithm, global optimization, tree-seed algorithm, reservoir production, industrial production

## Abstract

As the fields of engineering, energy, and geology become increasingly complex, decision makers face escalating challenges that require skilled solutions to meet practical production needs. Evolutionary algorithms, inspired by biological evolution, have emerged as powerful methods for tackling intricate optimization problems without relying on gradient data. Among these, the tree-seed algorithm (TSA) distinguishes itself due to its unique mechanism and efficient searching capabilities. However, an imbalance between its exploitation and exploration phases can lead it to be stuck in local optima, impeding the discovery of globally optimal solutions. This study introduces an improved TSA that incorporates water-cycling and quantum rotation-gate mechanisms. These enhancements assist the algorithm in escaping local peaks and achieving a more harmonious balance between its exploitation and exploration phases. Comparative experimental evaluations, using the CEC 2017 benchmarks and a well-known metaheuristic algorithm, demonstrate the upgraded algorithm’s faster convergence rate and enhanced ability to locate global optima. Additionally, its application in optimizing reservoir production models underscores its superior performance compared to competing methods, further validating its real-world optimization capabilities.

## 1. Introduction

Optimization problems have long been challenging and extensively researched subjects in scientific inquiry. These problems typically feature numerous suboptimal solutions and an escalating complexity, rendering the quest for optimal solutions increasingly daunting. Their prevalence in real-world scenarios necessitates continuous optimization efforts to enhance efficiency. In recent years, evolutionary algorithms have garnered significant interest and are progressively emerging as vital tools for addressing real-world challenges in the era of burgeoning big data [1,2]. Optimizing reservoir yield stands as a critical imperative within real-world industrial production. The primary goal is to pinpoint optimal solutions for individual wells, aimed at maximizing the net present value. Wang et al. [3] proposed an efficient and robust approach, called evolutionary-assisted reinforcement learning, for real-time production optimization under uncertainty. Specifically, we model the production optimization problem as a Markov decision process, where a reinforcement learning agent interacts with a reservoir simulator to train a control strategy aimed at maximizing a specified objective. Zhuang et al. [4] constructed a hybrid artificial intelligence approach to jointly optimize well location and well control parameters, taking into account both development objectives and dynamic optimization. A hybrid artificial intelligence approach combining a deep-learning method and a multi-objective optimization algorithm was built to find a compromise between the two. However, a combinatorial explosion of solution possibilities arises as the number of wells and production cycles increases, leading to larger dimensions of optimization variables. Consequently, this problem is categorized as a typical NP-hard problem, creating a favorable environment for the application of evolutionary algorithms.

In comparison to traditional mathematical methods, evolutionary algorithms are simple, efficient, and cost-effective, particularly when dealing with the complexities of current big data and parallel computing problems. The well-known evolutionary algorithms are differential evolution (DE) [5], ant-colony optimization (ACO) [6], and particle-swarm optimization (PSO) [7]. The more recently proposed novel evolutionary algorithms are the Kepler optimization algorithm (KOA) [8] and the coati optimization algorithm (COA) [9]. Many evolutionary algorithms draw inspiration from natural phenomena, achieving global optimal solutions through continuous iterative updates. For instance, the coati optimization algorithm simulates the behavior of coatis in nature, leveraging two primary behaviors: hunting and attacking iguanas and evading predators. Due to their unique advantages, evolutionary algorithms find widespread application across various fields. Zhao et al. [10] introduced an enhanced water-flow optimizer to optimize reservoir production by integrating a cross-search strategy to improve the convergence speed and accuracy of the algorithm. Song et al. [11] designed an adaptive collaborative co-evolutionary differential evolutionary algorithm with a dynamic hybrid mechanism of quantum evolutionary algorithm and genetic algorithm. The algorithm is also used to implement a late-train scheduling method for railroad trains to effectively eliminate the effects of late trains. Li et al. [12] improved the dung-beetle optimization and proposed a new improved evolutionary algorithm. The algorithm introduces a stochastic inverse learning strategy to improve population diversity and mitigate the problem of early convergence or local stagnation present in the algorithm. In addition, the proposed algorithm is applied to path-planning simulation experiments to generate shorter and faster paths, which effectively solves the practical application problems and demonstrates its potential for practical applications. Bian et al. [13] developed a discrete whale optimization algorithm for reducing uncorrelated variables and improving the prediction accuracy of a six-dollar edible blending oil sample. To validate the performance of the selected variables, partial least squares were used to model and predict the single oil content in hexane blended oils. Chen et al. [14] developed a 3D source-localization system using evolutionary algorithms in order to locate pollutant sources at unknown heights in a realistic indoor environment with weak airflow. Two 3D source-localization methods based on bionic swarm-intelligence algorithms, namely the 3D whale optimization algorithm and 3D particle-swarm optimization, were validated in unventilated indoor spaces of different heights. According to the no free lunch (NFL) [15] theory, one algorithm cannot always perform as the best on all Ops. Although the above algorithms show better results in specific applications, they do not focus on the search and exploration balance of the algorithms for specific problems, and the algorithms used do not have a simple search architecture, which is not conducive to optimization for specific problems.

The tree-seed algorithm (TSA) [16] is a recently proposed evolutionary algorithm. It draws inspiration from the reproductive process of trees, which propagate through seeds dispersed onto the ground. The TSA is characterized by its simplicity, ease of implementation, robust developmental capabilities, and promising application potential. With its innovative approach to population renewal, inspired by tree propagation methods, TSA has garnered significant attention from researchers. Babalik et al. [17] modify TSA appropriately; the proposed improved TSA is compared with other well-known optimization algorithms on 13 constrained optimization problems. The results show that the proposed algorithms have good performance in optimization quality and robustness. Aslan et al. [18] improved the update algorithm of TSA and proposed a new improved TSA. In the improved TSA, each dimension of a seed is updated independently and dynamically adjusted based on the operator dimension during seed creation. This approach provides significant advantages over TSA, particularly in handling large-scale benchmark functions. However, TSA’s development potential is limited by its simplistic division of population update modes into two cases, determined solely by the size of a random number. To address this limitation, this study introduces a water-cycle mechanism and quantum rotation-gate strategy into TSA, aiming to enhance its development capabilities. Comprehensive experiments and demonstrations have been conducted to validate the proposed enhancements. The performance of the proposed algorithm is compared against other well-known algorithms across 30 benchmark functions. Additionally, the algorithm is applied to real-world oil reservoir production scenarios, showcasing its practical utility. In summary, the main contributions of this study are outlined as follows:

- This study introduces an enhanced version of TSA, referred to as WQTSA, which incorporates the water-cycle mechanism and quantum rotation-gate strategy. These mechanisms enhance TSA’s global search capability and facilitate escaping local optima, thereby achieving a balance between search and exploration;
- To evaluate the effectiveness of the proposed WQTSA, it was benchmarked against other state-of-the-art evolutionary algorithms at IEEE CEC 2017. Additionally, this study offers a detailed analysis of how the two improvement mechanisms impact the performance of WQTSA, along with assessing its scalability across various dimensions;
- To assess the performance of the proposed WQTSA in addressing real production challenges, this study employs it to tackle the production optimization problem centered on three-channel reservoirs. Furthermore, the algorithm is compared against other state-of-the-art evolutionary algorithms in resolving this issue. The experimental findings underscore the outstanding optimization capabilities of the proposed algorithm in real-world scenarios.

The paper is structured as follows: Section 1 provides an overview of the motivation, background, and specific contributions of the study. Section 2 introduces the original TSA. Section 3 describes the introduced mechanisms and provides a detailed explanation of the proposed WQTSA. Section 4 presents all the results and an analysis obtained from the experiments. Finally, Section 5 concludes the study.

## 2. Overview of the Original TSA

Optimization involves finding the best solution among the possible solutions for a given problem based on specific criteria. It encompasses both maximization and minimization processes. If S represents the search space and F⊆S denotes the set of acceptable solutions within S, then f is the objective function. The goal of optimization is to find x∈F, as defined by the minimization and maximization processes in Equations (1) and (2), respectively.

(1) $$f\left(x\right)\leq f\left(y\right)\;\forall y\in F$$

(2) $$f\left(x\right)\geq f\left(y\right)\;\forall y\in F$$

Assuming up is the upper boundary of S and lp is the lower boundary of S, F needs to satisfy Equation (3).

(3) F=lp+rand(up−lp)

where rand is a random number within [0, 1].

If the function to be optimized is non-continuous, non-differentiable, or computationally expensive for high-dimensional search spaces, heuristic search methods can be employed to optimize it.

TSA is an evolutionary algorithm proposed by Kiran [16], which is inspired by the relationship between trees and seeds in nature. In nature, trees contact the ground through seeds, and then these seeds grow into new trees. TSA assumes that the ground is the search space, the trees and seeds are the candidate solutions, and the constantly generated seeds represent the emerging new candidate solutions. Updating Equation (4) reflects the use of the location of the tree, that is, the current optimal location to generate new seeds. Updating Equation (5) reflects the use of different positions of two trees to generate new seeds.

(4) $$S_{i,j}=T_{i,j}+\alpha_{i,j}\times \left(B_{j}-T_{r,j}\right)$$

(5) $$S_{i,j}=T_{i,j}+\alpha_{i,j}\times \left(T_{i,j}-T_{r,j}\right)$$

where $S_{i,j}$ represents the j-th dimension of the i-th seed that will be produced by the i-th tree. $T_{i,j}$ represents the j-th dimension of the i-th tree, $B_{j}$ represents the j-th dimension of the optimal tree obtained under the current iteration. The α is the scaling factor randomly produced in the range of [−1, 1]. 

The algorithm proposes two kinds of update equations, and choosing different update equations in different situations becomes the key to the algorithm. TSA designs a control parameter to control the algorithm to choose different update equations, which is called search tendency (ST) in the range of [0, 1]. In this study, referring to the TSA, the parameter ST is set to 0.1. The larger the value of ST, the stronger the local search ability of the TSA algorithm and the faster the algorithm converges to the optimal solution. The smaller the value of ST, the stronger the global search ability of the TSA algorithm. 

TSA initializes the position of the candidate solution through Equation (6), that is, the position of the initialization tree.

(6) $$T_{i,j}=L_{j,min}+r_{i,j}\times \left(H_{j,max}-L_{j,min}\right)$$

where $H_{j,max}$ is the upper bound of the search space and $L_{j,min}$ is the lower bound of the search space. The two variables that control the randomly generated candidate solution do not exceed the range of feasible solutions. The $r_{i,j}$ is a random number within [0, 1]. 

Similar to nature, TSA sets trees to generate seeds, not only to generate one seed but also to generate multiple seeds. The number of seeds depends on the size of the population. After the experiments, TSA set the minimum number of seeds to 10% of the population and the maximum number of seeds to 25% of the population size. The number generated is completely random within a reasonable range. 

**Figure 1:**
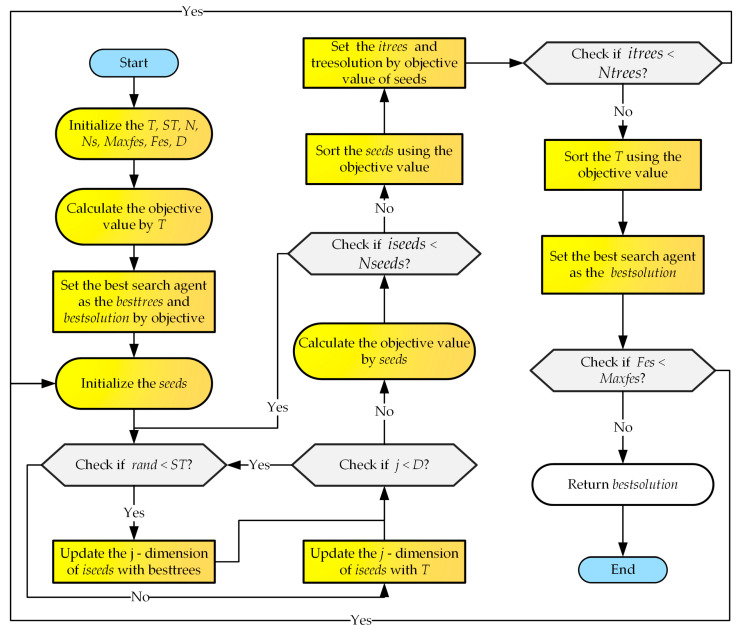
The flowchart of TSA.

The implementation of TSA is shown in Algorithm 1. The flowchart of TSA is shown in Figure 1. In Figure 1, *Fes* is the current number of iterations of the algorithm, and *Maxfes* is the maximum number of iterations. *iseeds* represents the individuals currently used in *S*. *itrees* represents the individuals currently used in *T*. *Nseeds* represents the total number of seed populations, and *Ntrees* represents the total number of tree populations.
**Algorithm 1**. Tree-seed algorithm**Input**: Search tendency *ST*, population size *N*, population dimension *D*.Initialize population *T* using Equation (6).**For** *T* Randomly generate the number of seeds to be between 10% and 25% of the total number of trees.**End For****For** the number of seeds** For** D**  IF** rand < *ST*   Update using Equation (4);**  Else**   Update using Equation (5);**  End IF**** End For****End For**Select the best seed of the current tree and compare it with this tree;Select the best solution of population *T*;**Return** best solution

## 3. The Proposed WQTSA

Inspired by the method of tree propagation, TSA has novel ideas and an ingenious design for population renewal, so it has been widely used by researchers. However, TSA only divides the update mode of the population into two cases, and the judgment standard is the size of a random number, which makes the exploration capability of TSA lacking. Therefore, in this study, the water-cycle mechanism and quantum rotation-gate strategy are introduced into TSA to enhance the exploration capability of the algorithm. 

### 3.1. The Water-Cycle Mechanism

The water-cycle mechanism is extracted from the water-cycle algorithm [19]. The idea of the water-cycle algorithm is very novel. Inspired by natural rivers, the population is divided into three levels: stream, river, and sea, according to the size of fitness. When the population is renewed, a stream flows into the river, and the river gathers into the sea. Finally, the water in the sea is recycled through evaporation and rainfall. After a continuous fitness value calculation and population circulation, the marine population with the best fitness value is finally obtained. The water-cycle algorithm has been applied in many fields by researchers because of its efficient performance. Korashy et al. [20] applied the improved algorithm of the water-cycle algorithm to the optimization model of the directional current relay to solve the optimal coordination problem. Osaba et al. [21] used the discrete version of the water-cycle algorithm to solve the traveling-salesman problem, including the symmetry problem and asymmetry problem. El-fergany et al. [22] used a water-cycle algorithm when coordinating overcurrent relays, and the advantages of the water-cycle algorithm are verified by simulation experiments. Elhameed et al. [23] optimize the single-objective and multi-objective economic load scheduling problem in the water-cycle algorithm. Its goal is the generating capacity of the unit, which improves the efficiency of the unit’s power scheduling. 

After the water-cycle mechanism was introduced into the TSA, it began to update the operation, mainly including confluence and evaporation rainfall. When the precipitation is completed, the stream is divided into two parts according to the fitness value. One part flows into the river and the other part flows into the sea. During this period, the location of the stream and river will be exchanged according to the fitness value. The specific update equation is shown in Equations (7) to (9).

(7) $$T_{Stream}^{i}=T_{Stream}^{i}+rand\times C\times \left(T_{Sea}^{i}-T_{Stream}^{i}\right)$$

(8) $$T_{Stream}^{i}=T_{Stream}^{i}+rand\times C\times \left(T_{River}^{i}-T_{Stream}^{i}\right)$$

(9) $$T_{River}^{i}=T_{River}^{i}+rand\times C\times \left(T_{Sea}^{i}-T_{River}^{i}\right)$$

where rand is a random number within 0–1, and *C* is a number within [0, 1], usually two. In the evaporation rainfall stage, we must first judge whether the evaporation conditions are met, that is, whether the distance between the river and the sea is less than the set threshold. The definition of this threshold determines the balance between concentration and evaporation of the algorithm. The equation definition is shown in the public notice.

(10) $$d_{max}^{i+1}=d_{max}^{i}-\frac{d_{max}^{i}}{maxit}$$

where maxit is the maximum number of iterations and $d_{max}^{i}$ is the parameter of adaptive adjustment, which decreases with the increase in the number of iterations. Once the evaporation conditions meet the requirements, the stream location will be updated to complete the evaporation rainfall process. The update equation is shown in Equation (11).

(11) $$T_{Stream}^{i,j}=L_{j,min}+r_{i,j}\times \left(H_{j,max}-L_{j,min}\right)$$

where $H_{j,max}$ is the upper bound of the search space and $L_{j,min}$ is the lower bound of the search space. The two variables that control the randomly generated candidate solution do not exceed the range of feasible solutions. The $r_{i,j}$ is a random number within [0, 1]. 

The implementation of the water-cycle mechanism is shown in Algorithm 2.
**Algorithm 2.** The water-cycle mechanism**Input:** Population *T* and fitness value *F*.According to the ranking of *F*, *T* is divided into *T_stream_*_,_ *T_river_*, *T_sea_*.Update the *T_stream_* by Equation (7);Update *T_stream_* and *F_stream_* and compare it with the *F_sea_*;Update the *T_stream_* by Equation (5);Calculate *F_river_* and compare it with *F_sea_*;Update *T_sea_* and *F_sea_* if there is a better one;Moving stream to river;Update the *T_stream_* by Equation (6);Calculate *F_stream_* and compare it with the *F* of the *F_river_*;Update *T_river_* and *F_river_* if there is a better one;Update *T_stream_* based on evaporation conditions and raining process;**Return** *T*;

### 3.2. The Quantum Rotation-Gate Strategy

The concept of quantum rotation gate first appeared in physics a century ago and has been introduced into different fields [24]. In WQTSA, we introduce a quantum rotation-gate strategy to enhance the diversity of the algorithm, so as to enhance the global search ability of the algorithm. In the swarm-intelligence optimization algorithm, the data, when the group is updated, are generally floating-point numbers, while quantum generally uses discrete data. Therefore, these values are first designed to be converted, and the conversion process is shown in Equations (12) and (13).

(12) $$U\left(\theta_{i}\right)=\left[\begin{array}{cc} cos\left(\theta_{i}\right) & -sin\left(\theta_{i}\right)\\ sin\left(\theta_{i}\right) & cos\left(\theta_{i}\right) \end{array}\right]$$

(13) $$\left[\begin{array}{c} \alpha_{i}\\ \beta_{i} \end{array}\right]=U\left(\theta_{i}\right)\left[\begin{array}{c} \alpha_{i}\\ \beta_{i} \end{array}\right]=\left[\begin{array}{cc} cos\left(\theta_{i}\right) & -sin\left(\theta_{i}\right)\\ sin\left(\theta_{i}\right) & cos\left(\theta_{i}\right) \end{array}\right]\left[\begin{array}{c} \alpha_{i}\\ \beta_{i} \end{array}\right]$$

where $\theta_{i}$, denoting the i−th rotation angle, its direction of rotation, and size, is set in advance. In addition, the adjustment method of $\theta_{i}$ in WQTSA is shown in Table 1. ${\left(\alpha_{i},\beta_{i}\right)}^{T}$ and ${\left(\alpha_{i}^{\prime },\beta_{i}^{\prime }\right)}^{T}$ represent the quantum bit state vectors before and after the rotation-gate update of the i-th quantum bit of an individual within a population. 

**Algorithm 3**. The quantum rotation-gate strategy
**Input**: *α*, *β*, rotation degree *s*, rotation angle delta, position *T* and fitness values of *T*, dim;**For** *i* = 1*:N*** For** *j* = 1:*Dim*  Update *α*, *β*, delta and *s*;  Compare the fitness (*i*) and *best_fitness*;  Update the delta and *s* according to Table 1;  Perform quantum rotation gate strategy by Equation (11) to obtain updated *T*;** End** ForCalculate the fitness;**End** For**Return** *T*


### 3.3. The Proposed WQTSA

**Figure 2:**
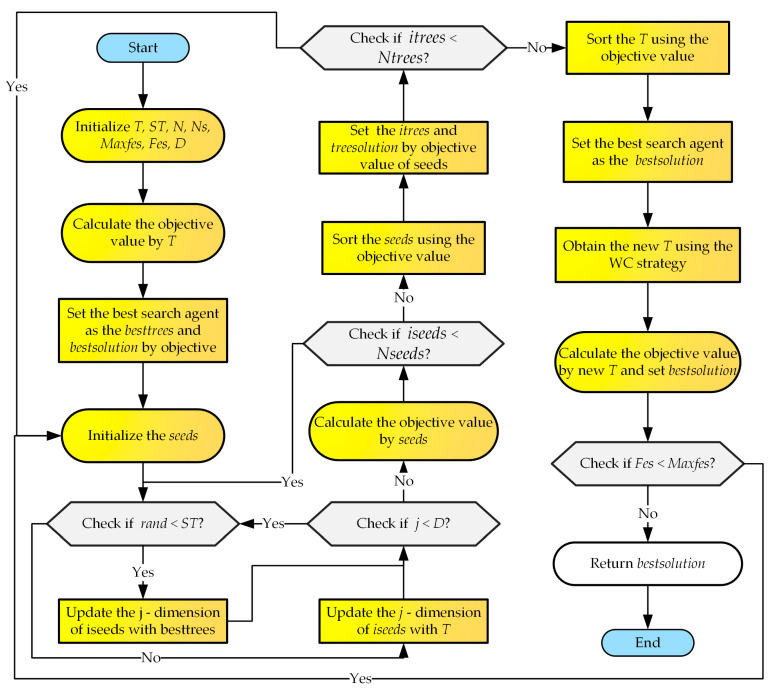
Flowchart of WQTSA.

This section adds the two ideas mentioned above to the TSA algorithm and proposes a new WQTSA. WQTSA continues to further develop the current population after TSA’s update operator. The algorithm first uses a quantum rotation-gate strategy to update the population and, then, uses a water-cycle mechanism to further develop. The implementation of WQTSA is in Algorithm 4. The flowchart of WQTSA is shown in Figure 2.
**Algorithm 4**. The proposed WQTSA**Input**: Search tendency *ST*, population size *N*, population dimension *D*.Initialize population *T* using Equation (6).**For**  *T* Randomly generate the number of seeds to be between 10% and 25% of the total number of trees**End  For****For** the number of seeds** For** D**  IF** rand < *ST*   Update using Equation (4);**  Else**   Update using Equation (5);**  End IF**** End For****End For**Update the *T* using quantum rotation gate strategy;Update the *T* using water cycle mechanism;Select the best seed of the current tree and compare it with this tree;Select the best solution of population *T*;**Return** best solution

Figure 2 shows the flowchart of WQTSA. The time complexity of WQTSA is determined by the water-cycle mechanism, quantum rotation-gate strategy, initialization, and iterative updates of the TSA. The main parameters are the population size (n), the iterations (i), the number of seeds (ns), and the dimension (d). Therefore, the time complexity of WQTSA is *O* (WQTSA) = *O* (initialization) + *O* (Population update) + *O* (water-cycle mechanism) + *O* (quantum rotation-gate strategy) ≈ *O (*n×d) + *O* (n×d×i×ns) + *O* (n) + *O* (n×d) ≈ *O* (i×d×n×ns).

## 4. Experimental Results and Analysis

The performance of WQTSA will be critically evaluated in several ways. First, in the 30 test functions of IEEE CEC 2017, the multi-mechanism enhancement effect and multi-dimensional scalability of WQTSA will be analyzed. Second, WQTSA will be compared with state-of-the-art evolutionary algorithms. The IEEE CEC 2017 test functions are commonly used to test the performance of comparative optimization algorithms. There are 30 functions categorized into single-peak, hybrid, combined, and multi-peak functions. The parameter settings of the algorithm in the experiment are shown in Table 2. In order to test the optimization performance of WQTSA in real problems, it is applied to the optimization of reservoir production.

### 4.1. Experiments on Benchmark Test Functions

#### 4.1.1. Strategy Validation

The WQTSA comprises a foundational TSA and two enhanced mechanisms, namely the WS (water cycle) and the quantum rotation-gate strategy. In this section, we aim to assess the impact of these two mechanisms on TSA and explore their individual contributions to the enhancement of TSA. Crucially, we will illustrate that the two mechanisms in WQTSA synergistically reinforce each other, emphasizing that the absence of any one mechanism prevents achieving the optimal performance of WQTSA. 

Table 3 records the mechanisms for adding different improved TSA, namely WQTSA, WTSA, QTSA, and TSA. The ‘1’ in the table represents participating in the mechanism. The ‘0’ represents that no mechanism has been added. From Table 3, it can be seen that WQTSA has added two mechanisms. WTSA has added WS. QTSA has added a quantum rotation gate. TSA has not added any mechanism.

Table 4 presents the experimental results of multi-mechanism analysis for WQTSA. ‘Rank’ signifies the algorithm’s position, ‘AVG’ represents its average ranking, and the symbols ‘+/−/=’ denote whether the WQTSA outperforms, underperforms, or equals other algorithms. From Table 4, the ranking of the eight variants from good to bad is WQTSA > WTSA > QTSA > TSA. WQTSA ranked first among all variants. All variants have better performance than the original TSA, indicating that all three improvement mechanisms have an enhancing effect on TSA. WTSA ranks second, indicating that the water-cycle mechanism has greatly improved TSA. But with the addition of the quantum rotation-gate strategy, WQTSA has achieved even greater improvement.

#### 4.1.2. Scalability Test

This section tests the performance of WQTSA in different dimensions, including 50 and 100 dimensions. In modern computing systems, multi-dimensional scalability testing is crucial for assessing system performance under diverse real-world conditions. By evaluating scalability across multiple dimensions, such as workload, resources, and environmental factors, stakeholders gain insights into system behavior and identify optimization opportunities. This comprehensive approach enables the design of more robust and efficient systems capable of meeting evolving demands. The comparison algorithms in the experiment include WQTSA and TSA. Except for different dimensions, other parameters and environment settings of the two algorithms are consistent. The specific experimental results are recorded in Table 5. It can be seen from the data in the table that WQTSA is superior to TSA, not only in 50 dimensions but also in 100 dimensions. This shows that WQTSA still has strong stability and scalability in the face of different complex problems.

#### 4.1.3. Comparison with Other Algorithms

In this section, WQTSA is compared with other meta-heuristic algorithms on CEC2017. The compared algorithms include HGWO [25], LGMSFOA, CGSCA [26], CCMWOA [27], m_SCA [28], SCADE [29], WEMFO [30], DE, MVO, SSA [31,32], and TSA. The experimental results of the algorithm comparison are recorded in Table 6. In order to maintain the accuracy of the experiment, there are 30 iterations in total. Std in the table is the standard deviation of multiple iterations, and Avg is the average value of multiple iterations. The last column of the table also records the ranking of the algorithm, where ‘+/−/=’ represents the number of times WQTSA is better than, worse than, and equal to other comparison algorithms in 30 iterations. In the 30-iteration experiment, the population size of all the algorithms was set to 30, the population dimension was set to 30, and the number of evaluations was set to 300,000. For the other parameters, refer to the original papers for each comparison algorithm.

As can be seen from Table 6, WQTSA ranks first in all comparison algorithms and is superior to HGWO, LGMSFOA, CGSCA, CCMWOA, and SCADE in all test functions. WQTSA is superior to m_SCA in 27 functions. WQTSA is superior to WEMFO in 28 functions. WQTSA is superior to DE in 17 functions. WQTSA is superior to PSO in 26 functions. WQTSA is superior to SMA in 25 functions. WQTSA is superior to TSA in 23 functions. 

The experimental *p*-value information is recorded in Table 7. In scientific research, the *p*-value serves as a crucial statistical measure used to evaluate the strength of evidence against a null hypothesis. It represents the probability of obtaining the observed results, or more extreme results, under the assumption that the null hypothesis is true. A low *p*-value indicates that the observed results are unlikely to occur if the null hypothesis is true, thus providing evidence against the null hypothesis. Typically, a significance level (alpha) is chosen beforehand to determine the threshold for rejecting the null hypothesis. If the *p*-value is less than or equal to the significance level, the null hypothesis is rejected, suggesting that the observed results are statistically significant. Conversely, if the *p*-value is greater than the significance level, there is insufficient evidence to reject the null hypothesis. It is important to note that, while a small *p*-value indicates statistical significance, it does not necessarily imply the practical significance or importance of the observed effect. Therefore, the interpretation of *p*-values should be considered in conjunction with the effect size and practical relevance when drawing conclusions from research findings. In Table 7, most of the data are less than 0.05. A *p*-value less than 0.05 shows that the experimental results are relatively reliable and not accidental. 

**Figure 3:**
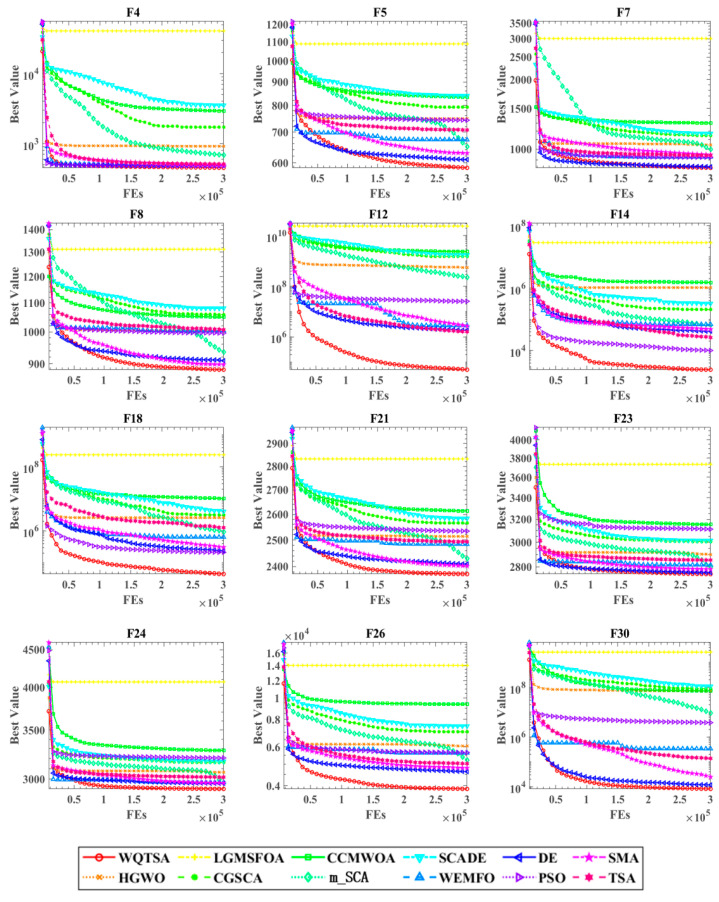
Comparison of convergence curves between WQTSA and other well-known optimization methods on IEEE CEC 2017 test functions.

Figure 3 shows the convergence curve of the proposed algorithm with other state-of-the-art algorithms in the dataset. Convergence analysis plays a pivotal role in assessing the performance of evolutionary algorithms in solving optimization problems. The convergence curve, a fundamental metric in EA analysis, illustrates the behavior of the algorithm’s objective function value over successive iterations. It serves as a visual representation of the algorithm’s progress towards finding optimal or near-optimal solutions. The convergence curve typically exhibits a decreasing trend, reflecting the algorithm’s gradual improvement in optimizing the objective function. The rate of convergence, indicated by the steepness of the curve, provides insights into the algorithm’s efficiency in exploring the search space and exploiting promising solutions. Moreover, convergence analysis enables researchers to identify potential convergence criteria, such as a predefined tolerance level or a maximum number of iterations, to determine when the algorithm has sufficiently converged to a solution. On the way, it can be seen more clearly that WQTSA can converge faster and obtain a smaller fitness value than other comparison algorithms. This also shows that WQTSA is effective in improving TSA. 

### 4.2. Application to Oil Reservoir Production

The objective of optimizing reservoir production is to identify optimal solutions for each well, with the aim of maximizing the net present value (NPV). However, a combinatorial explosion of solution possibilities arises, as the number of wells and production cycles increases, leading to larger dimensions of optimization variables. Consequently, this problem is categorized as a typical NP-hard problem, creating a favorable environment for the application of evolutionary algorithms. 

In this section, we employ WQTSA within the context of a three-channel reservoir model using the reservoir numerical simulation software Eclipse. We then proceed to assess the performance of WQTSA by comparing it with several classical evolutionary algorithms. In our experimental setup, we simplify the complexity by neglecting the nonlinear constraints present in oilfield production. Instead, we focus on optimizing the objective function, specifically the net present value (NPV), as detailed in Equation (14).

(14) $$NPV\left(x,z\right)=\sum_{t=1}^{n}\;\Delta t\frac{Q_{o,t}\cdot r_{o}-Q_{w,t}\cdot r_{w}-Q_{i,t}\cdot r_{i}}{{\left(1+b\right)}^{p_{t}}}$$

where x represents the set of variables subject to optimization, and where, in this experiment, these variables correspond to the injection and recovery rates of each well. The variable z serves as the state parameter of the model, describing the construction of the numerical reservoir model. n denotes the total simulation time, and $Q_{o,t}$, $Q_{w,t}$, $Q_{i,t}$ represent the water production rate, oil production rate, and water injection rate, respectively, at time step t. $r_{o}$ signifies the oil revenue, and $r_{w}$ and $r_{i}$ denote the cost of treating and injecting water, respectively. b represents the average annual interest rate, and $p_{t}$ indicates the number of years elapsed. 

**Figure 4:**
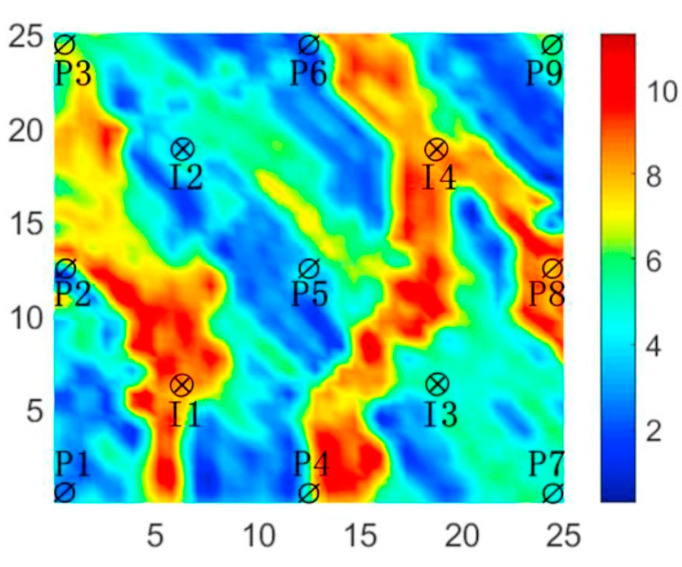
Log-permeability distribution of three-channel model.

The three-channel reservoir model is characterized as a non-homogeneous two-dimensional reservoir, with nine production wells and four injection wells arranged in a five-point pattern. The model is represented by a grid with dimensions of 25 × 25 × 1, where each grid block measures 100 ft in length. Additionally, each grid block has a thickness of 20 ft, and the porosity is uniformly set at 0.2. The specific distribution of the modeled permeability is illustrated in Figure 4. 

The optimization variables in this production optimization problem include the injection rate of each injection well and the fluid recovery rate of the production well. The water injection rate ranges from 0 to 500 STB/DAY, and the water extraction rate for production wells ranges from 0 to 200 STB/DAY. The thermal storage has a usage time of 1800 days, and the decision time step is set to 360 days. Consequently, the dimensionality of the decision variable is 65. The fitness function for optimization is the NPV, where the oil price is fixed at 80.0 USD/STB, the water injection cost is 5.0 USD/STB, and the water treatment cost is 5.0 USD/STB. For simplicity, the model assumes an average annual interest rate of 0%. 

To demonstrate the effectiveness of the algorithm improvement, WQTSA is optimized simultaneously with TSA, DE, and PSO to compare the model. To ensure experimental fairness, all optimizations are conducted 10 times, and the average of the last NPV obtained is considered for analysis. 

**Figure 5:**
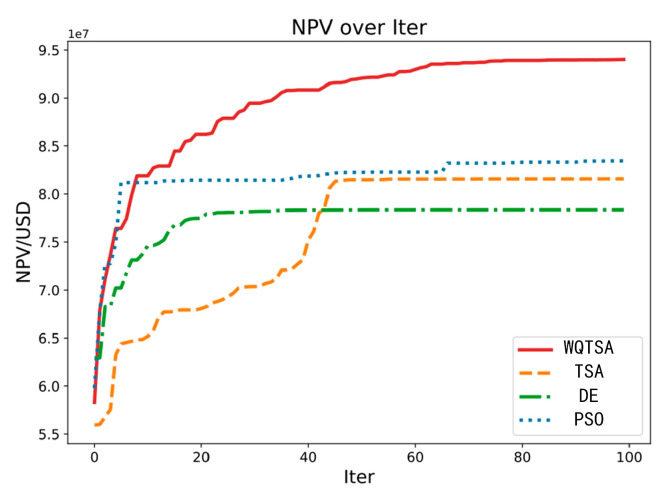
NPV obtained by the algorithms with iteration.

Figure 5 illustrates the optimal NPV values obtained by four methods against the number of iterations. The red line represents WQTSA, the blue line represents PSO, the yellow line represents TSA, and the green line represents DE. Observing the figure, it is evident that WQTSA demonstrates notable superiority over PSO, TSA, and DE, consistently achieving higher NPV values within the same number of iterations. 

**Figure 6:**
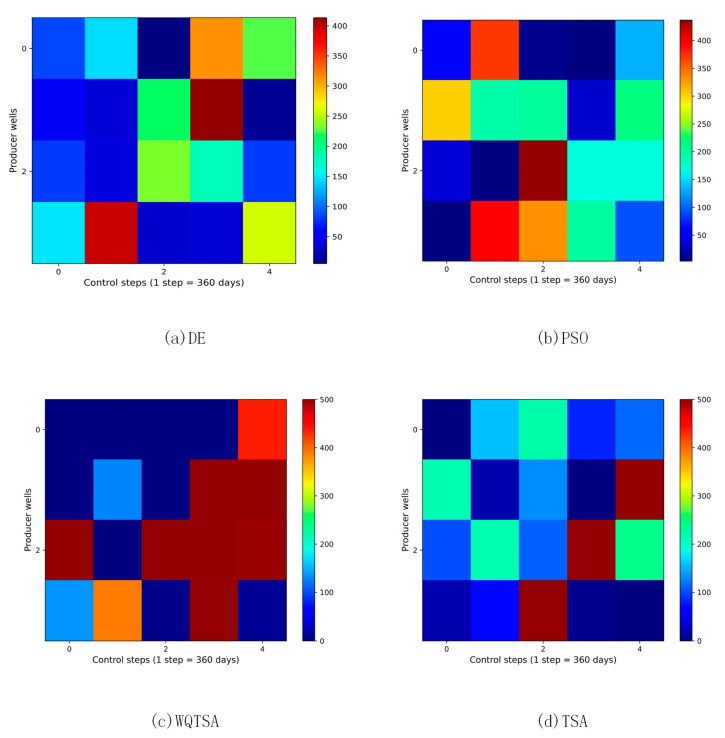
The optimal water injection rate obtained by each algorithm for the three-channel model.

**Figure 7:**
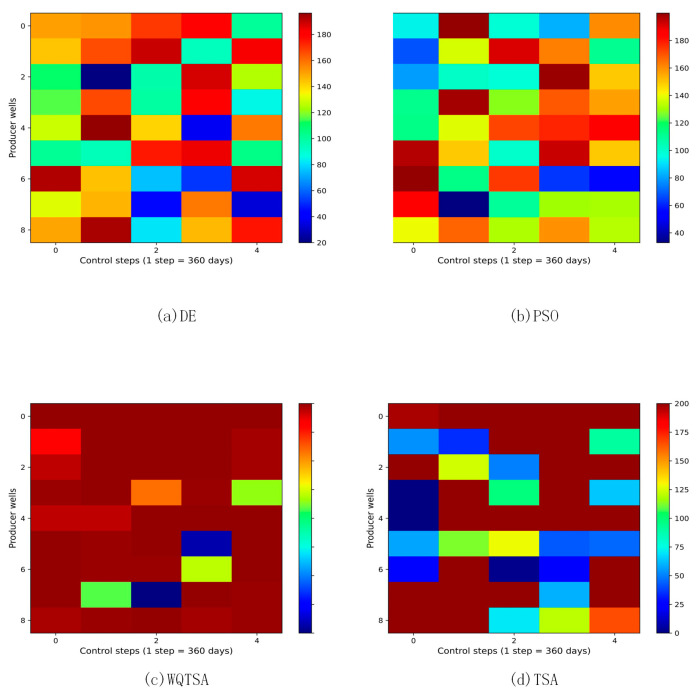
The optimal liquid production rate obtained by each algorithm for the three-channel model.

The final optimization schemes for the water injection rate and the liquid production rate for the four methods are depicted in Figure 6 and Figure 7. In these figures, the horizontal axis represents the practice step size, and the vertical axis represents the well number.

In Figure 6, the regulation scheme for the injection wells of TSA is displayed, revealing significant variations in the injection rates of the same wells at the adjacent control step values. This instability is not conducive to field implementation, and the fluctuating injection rates may adversely impact bottomhole pressure, potentially damaging the reservoir and hindering sustainable development. In contrast, WQTSA provides a smoother production scheme compared to TSA, as demonstrated in the figures. Figure 7 shows the liquid production speed for the four algorithms, and it can be seen that WQTSA has a clear advantage over the other three algorithms.

## 5. Conclusions

In this study, we introduce WQTSA, which integrates a water-cycle mechanism and a quantum rotation-gate strategy to enhance the effectiveness of TSA. The water-cycle mechanism accelerates algorithm convergence by reducing the random rate and increasing the step size. Simultaneously, the quantum rotation-gate strategy enhances global exploration by facilitating information exchange among individuals, thereby increasing the likelihood of avoiding local optima.

We systematically compare the proposed WQTSA with algorithms, advanced algorithms, and TSA variants utilizing IEEE CEC 2017 benchmark functions. Empirical results illustrate that WQTSA surpasses other optimizers in both convergence speed and solution accuracy across diverse benchmark functions. Furthermore, the suggested approach is implemented on a reservoir model with three channels and is contrasted with three algorithms. The results indicate that WQTSA has the capability to achieve increased NPV values while maintaining the same number of assessments. It emerges as a compelling alternative for optimizing reservoir production. 

In future endeavors, our focus will be on further enhancing WQTSA to mitigate algorithmic time costs while concurrently improving optimization performance. Additionally, we plan to extend the application of WQTSA to diverse scenarios. 

## Figures and Tables

**Table 1:** The selection strategy of rotation angle in quantum rotation-gate strategy.

	${∆\theta }_{i}$	${s(\alpha }_{j},\beta_{j})$ $\alpha_{j}\beta_{j}>0$	$\alpha_{j}\beta_{j}<0$	$\alpha_{j}=0$	$\beta_{i}=0$
$f(x_{i})=best\_fitness$ $f\left(x_{i}\right)>best\_fitness$	δ	0 +1	0 −1	0 ±1	0 0
$f\left(x_{i}\right)<best\_fitness$		−1	+1	0	±1

where $f(x_{i})$ indicates the fitness of the i−th population, best_fitness indicates the best fitness, δ indicates the angle of rotation, ${s(\alpha }_{j},\beta_{j})$ indicates the direction of rotation, $\alpha_{j}$ indicates the individual value for the j−th position in the corresponding population, and $\beta_{j}$ represents the individual value of the j-th position in the corresponding population. The main role of quantum rotation-gate strategy in TSA is to determine the excellent population by comparing the two populations and transferring the inferior population to the dominant population.

**Table 2:** Parameters.

Population Size	Dimension	Maximum Evaluation
30	30	300,000

**Table 3:** Various TSAs from two mechanisms.

Algorithm	Water-Cycle Mechanism	Quantum Rotation-Gate Strategy
WQTSA	1	1
WTSA	1	0
QTSA	0	1
TSA	0	0

**Table 4:** Experimental results of multi-mechanism analysis for WQTSA.

Algorithm	Rank	+/=/−	AVG
WQTSA	1	~	1.7511
WTSA	2	2/28/0	1.8055
QTSA	3	14/12/4	3.1644
TSA	4	16/10/4	3.2789

**Table 5:** Multidimensional test results of WQTSA and TSA on IEEE CEC 2017 test functions.

Function	Method	Dim = 50		Dim = 100	
		Avg	Std	Avg	Std
F1	WQTSA	1.4197 × 10^3^	1.4471 × 10^3^	4.2093 × 10^3^	3.1504 × 10^3^
	TSA	2.1062 × 10^4^	2.3542 × 10^4^	4.7241 × 10^9^	1.1577 × 10^9^
F2	WQTSA	3.9106 × 10^35^	1.8553 × 10^36^	6.0307 × 10^102^	3.3017 × 10^103^
	TSA	5.1592 × 10^56^	1.3834 × 10^57^	1.7191 × 10^137^	7.6164 × 10^137^
F3	WQTSA	1.8549 × 10^4^	8.5976 × 10^3^	1.0695 × 10^5^	2.8537 × 10^4^
	TSA	1.2177 × 10^5^	1.4129 × 10^4^	3.6799 × 10^5^	2.9832 × 10^4^
F4	WQTSA	5.0997 × 10^2^	4.9222 × 10	6.5064 × 10^2^	3.9680 × 10
	TSA	6.8107 × 10^2^	3.6427 × 10	2.5319 × 10^3^	7.2137 × 10^2^
F5	WQTSA	6.6928 × 10^2^	4.4114 × 10	9.6846 × 10^2^	1.0564 × 10^2^
	TSA	9.3416 × 10^2^	1.9242 × 10	1.5977 × 10^3^	2.7811 × 10
F6	WQTSA	6.0005 × 10^2^	2.7953 × 10^−2^	6.1336 × 10^2^	7.9760
	TSA	6.0127 × 10^2^	3.5299 × 10^−1^	6.2336 × 10^2^	2.5496
F7	WQTSA	9.9324 × 10^2^	8.0259 × 10	1.4958 × 10^3^	1.7223 × 10^2^
	TSA	1.2151 × 10^3^	2.2312 × 10	2.1728 × 10^3^	6.0895 × 10
F8	WQTSA	9.7020 × 10^2^	5.7475 × 10	1.3352 × 10^3^	1.2678 × 10^2^
	TSA	1.2382 × 10^3^	1.5723 × 10	1.9065 × 10^3^	3.2687 × 10
F9	WQTSA	1.2881 × 10^3^	2.4520 × 10^2^	1.5701 × 10^4^	5.4030 × 10^3^
	TSA	2.1181 × 10^3^	4.1122 × 10^2^	2.5347 × 10^4^	3.4631 × 10^3^
F10	WQTSA	1.0590 × 10^4^	2.3092 × 10^3^	2.2647 × 10^4^	4.7890 × 10^3^
	TSA	1.4207 × 10^4^	3.6583 × 10^2^	3.1020 × 10^4^	4.1899 × 10^2^
F11	WQTSA	1.2469 × 10^3^	4.2471 × 10	2.1120 × 10^3^	2.6483 × 10^2^
	TSA	1.4328 × 10^3^	3.4992 × 10	3.9925 × 10^4^	6.2341 × 10^3^
F12	WQTSA	6.2668 × 10^5^	2.9751 × 10^5^	2.2766 × 10^6^	8.1725 × 10^5^
	TSA	5.0190 × 10^7^	1.8423 × 10^7^	9.1039 × 10^8^	3.2296 × 10^8^
F13	WQTSA	3.1540 × 10^3^	2.9128 × 10^3^	4.8837 × 10^3^	2.6779 × 10^3^
	TSA	3.4657 × 10^3^	2.4187 × 10^3^	5.0222 × 10^3^	1.7070 × 10^3^
F14	WQTSA	2.0172 × 10^4^	2.0150 × 10^4^	2.9168 × 10^5^	2.5950 × 10^5^
	TSA	2.9488 × 10^5^	1.8088 × 10^5^	1.1044 × 10^7^	2.9593 × 10^6^
F15	WQTSA	9.1881 × 10^3^	5.6308 × 10^3^	2.7625 × 10^3^	9.6328 × 10^2^
	TSA	7.3885 × 10^3^	4.0376 × 10^3^	2.6287 × 10^3^	6.6934 × 10^2^
F16	WQTSA	3.5016 × 10^3^	4.0734 × 10^2^	6.8120 × 10^3^	1.1096 × 10^3^
	TSA	4.7224 × 10^3^	2.4342 × 10^2^	1.0137 × 10^4^	3.0764 × 10^2^
F17	WQTSA	3.0127 × 10^3^	3.4188 × 10^2^	5.3166 × 10^3^	7.3976 × 10^2^
	TSA	3.6998 × 10^3^	1.1478 × 10^2^	6.9918 × 10^3^	2.3810 × 10^2^
F18	WQTSA	1.9738 × 10^5^	1.7608 × 10^5^	5.9470 × 10^5^	3.9607 × 10^5^
	TSA	6.3561 × 10^6^	1.9752 × 10^6^	2.2474 × 10^7^	5.9624 × 10^6^
F19	WQTSA	1.8234 × 10^4^	5.6302 × 10^3^	3.3909 × 10^3^	1.1063 × 10^3^
	TSA	1.4543 × 10^4^	4.3221 × 10^3^	2.9141 × 10^3^	7.7287 × 10^2^
F20	WQTSA	3.1654 × 10^3^	2.9736 × 10^2^	5.5297 × 10^3^	6.5849 × 10^2^
	TSA	3.6917 × 10^3^	1.6412 × 10^2^	7.0047 × 10^3^	1.7127 × 10^2^
F21	WQTSA	2.4701 × 10^3^	6.4127 × 10	2.8205 × 10^3^	1.1717 × 10^2^
	TSA	2.7273 × 10^3^	1.1603 × 10	3.4304 × 10^3^	2.8141 × 10
F22	WQTSA	6.0001 × 10^3^	4.8530 × 10^3^	2.5513 × 10^4^	4.8490 × 10^3^
	TSA	9.7356 × 10^3^	5.6897 × 10^3^	3.3378 × 10^4^	4.4458 × 10^2^
F23	WQTSA	2.9446 × 10^3^	5.8987 × 10	3.3266 × 10^3^	1.6564 × 10^2^
	TSA	3.1744 × 10^3^	1.9795 × 10	3.9338 × 10^3^	1.0051 × 10^2^
F24	WQTSA	3.1068 × 10^3^	6.9419 × 10	3.9542 × 10^3^	1.8350 × 10^2^
	TSA	3.3311 × 10^3^	2.1892 × 10	4.5084 × 10^3^	5.2372 × 10
F25	WQTSA	3.0753 × 10^3^	2.4312 × 10	3.3198 × 10^3^	3.8803 × 10
	TSA	3.1299 × 10^3^	2.1999 × 10	5.0026 × 10^3^	5.8189 × 10^2^
F26	WQTSA	6.2454 × 10^3^	9.6312 × 10^2^	1.4683 × 10^4^	2.7080 × 10^3^
	TSA	8.1255 × 10^3^	1.7772 × 10^2^	1.8064 × 10^4^	4.4427 × 10^2^
F27	WQTSA	3.4545 × 10^3^	1.2042 × 10^2^	3.6726 × 10^3^	1.0113 × 10^2^
	TSA	3.6599 × 10^3^	6.2266 × 10	4.4969 × 10^3^	3.0731 × 10^2^
F28	WQTSA	3.3041 × 10^3^	1.6297 × 10	3.4165 × 10^3^	3.8194 × 10
	TSA	3.3841 × 10^3^	3.4199 × 10	5.4609 × 10^3^	6.6265 × 10^2^
F29	WQTSA	4.3791 × 10^3^	3.5064 × 10^2^	7.9001 × 10^3^	8.6866 × 10^2^
	TSA	5.1581 × 10^3^	2.7747 × 10^2^	9.9630 × 10^3^	3.6747 × 10^2^
F30	WQTSA	1.0761 × 10^6^	6.7357 × 10^5^	1.8462 × 10^4^	1.0874 × 10^4^
	TSA	3.7897 × 10^6^	1.3978 × 10^6^	1.1488 × 10^6^	3.8097 × 10^5^

**Table 6:** Comparative experimental ranking results of WQTSA and other famous optimization methods on IEEE CEC 2017 test functions.

Algorithm	Rank	+/=/−	AVG
WQTSA	1	~	1.8667
HGWO	8	30/0/0	7.9667
LGMSFOA	12	30/0/0	12.0000
CGSCA	9	30/0/0	8.9000
CCMWOA	11	30/0/0	10.2000
m_SCA	6	27/2/1	5.5333
SCADE	10	30/0/0	10.0333
WEMFO	5	28/1/1	5.36667
DE	2	17/8/5	2.4333
PSO	7	26/2/2	5.6333
SMA	3	25/1/4	3.6000
TSA	4	23/1/6	4.4007

**Table 7:** The *p*-values of WQTSA versus other algorithms.

	HGWO	LGMSFOA	CGSCA	CCMWOA	m_SCA	SCADE
F1	1.7344 × 10^−6^	1.7344 × 10^−6^	1.7344 × 10^−6^	1.7344 × 10^−6^	1.7344 × 10^−6^	1.7344 × 10^−6^
F2	1.7344 × 10^−6^	1.7344 × 10^−6^	1.7344 × 10^−6^	1.7344 × 10^−6^	1.7344 × 10^−6^	1.7344 × 10^−6^
F3	1.7344 × 10^−6^	1.7344 × 10^−6^	1.7344 × 10^−6^	1.7344 × 10^−6^	1.7344 × 10^−6^	1.7344 × 10^−6^
F4	1.7344 × 10^−6^	1.7344 × 10^−6^	1.7344 × 10^−6^	1.7344 × 10^−6^	1.7344 × 10^−6^	1.7344 × 10^−6^
F5	1.7344 × 10^−6^	1.7344 × 10^−6^	1.7344 × 10^−6^	1.7344 × 10^−6^	1.7344 × 10^−6^	1.7344 × 10^−6^
F6	1.7344 × 10^−6^	1.7344 × 10^−6^	1.7344 × 10^−6^	1.7344 × 10^−6^	1.7344 × 10^−6^	1.7344 × 10^−6^
F7	1.7344 × 10^−6^	1.7344 × 10^−6^	1.7344 × 10^−6^	1.7344 × 10^−6^	1.9209 × 10^−6^	1.7344 × 10^−6^
F8	1.7344 × 10^−6^	1.7344 × 10^−6^	1.7344 × 10^−6^	1.7344 × 10^−6^	1.6394 × 10^−5^	1.7344 × 10^−6^
F9	1.7344 × 10^−6^	1.7344 × 10^−6^	1.7344 × 10^−6^	1.7344 × 10^−6^	1.7344 × 10^−6^	1.7344 × 10^−6^
F10	2.1827 × 10^−2^	1.7344 × 10^−6^	1.9209 × 10^−6^	7.7122 × 10^−4^	4.3896 × 10^−3^	1.7344 × 10^−6^
F11	1.7344 × 10^−6^	1.7344 × 10^−6^	1.7344 × 10^−6^	1.7344 × 10^−6^	1.7344 × 10^−6^	1.7344 × 10^−6^
F12	1.7344 × 10^−6^	1.7344 × 10^−6^	1.7344 × 10^−6^	1.7344 × 10^−6^	1.7344 × 10^−6^	1.7344 × 10^−6^
F13	1.7344 × 10^−6^	1.7344 × 10^−6^	1.7344 × 10^−6^	1.7344 × 10^−6^	1.7344 × 10^−6^	1.7344 × 10^−6^
F14	1.7344 × 10^−6^	1.7344 × 10^−6^	1.7344 × 10^−6^	1.7344 × 10^−6^	1.7344 × 10^−6^	1.7344 × 10^−6^
F15	1.7344 × 10^−6^	1.7344 × 10^−6^	1.7344 × 10^−6^	1.7344 × 10^−6^	1.7344 × 10^−6^	1.7344 × 10^−6^
F16	1.7344 × 10^−6^	1.7344 × 10^−6^	1.7344 × 10^−6^	1.9209 × 10^−6^	2.3038 × 10^−2^	1.7344 × 10^−6^
F17	1.7344 × 10^−6^	1.7344 × 10^−6^	1.7344 × 10^−6^	1.7344 × 10^−6^	7.5213 × 10^−2^	1.7344 × 10^−6^
F18	1.7344 × 10^−6^	1.7344 × 10^−6^	1.7344 × 10^−6^	1.7344 × 10^−6^	1.7344 × 10^−6^	1.7344 × 10^−6^
F19	1.7344 × 10^−6^	1.7344 × 10^−6^	1.7344 × 10^−6^	1.7344 × 10^−6^	1.9209 × 10^−6^	1.7344 × 10^−6^
F20	1.7344 × 10^−6^	1.7344 × 10^−6^	2.1266 × 10^−6^	5.2165 × 10^−6^	4.9498 × 10^−2^	1.7344 × 10^−6^
F21	1.7344 × 10^−6^	1.7344 × 10^−6^	1.7344 × 10^−6^	1.7344 × 10^−6^	5.7517 × 10^−6^	1.7344 × 10^−6^
F22	1.7344 × 10^−6^	1.7344 × 10^−6^	1.7344 × 10^−6^	1.7344 × 10^−6^	1.7344 × 10^−6^	1.7344 × 10^−6^
F23	1.7344 × 10^−6^	1.7344 × 10^−6^	1.7344 × 10^−6^	1.7344 × 10^−6^	1.1265 × 10^−5^	1.7344 × 10^−6^
F24	1.7344 × 10^−6^	1.7344 × 10^−6^	1.7344 × 10^−6^	1.7344 × 10^−6^	1.4936E-05	1.7344 × 10^−6^
F25	1.7344 × 10^−6^	1.7344 × 10^−6^	1.7344 × 10^−6^	1.7344 × 10^−6^	1.7344 × 10^−6^	1.7344 × 10^−6^
F26	1.7344 × 10^−6^	1.7344 × 10^−6^	1.7344 × 10^−6^	1.7344 × 10^−6^	6.3391 × 10^−6^	1.7344 × 10^−6^
F27	1.7344 × 10^−6^	1.7344 × 10^−6^	1.7344 × 10^−6^	1.7344 × 10^−6^	3.3173 × 10^−4^	1.7344 × 10^−6^
F28	1.7344 × 10^−6^	1.7344 × 10^−6^	1.7344 × 10^−6^	1.7344 × 10^−6^	1.7344 × 10^−6^	1.7344 × 10^−6^
F29	1.7344 × 10^−6^	1.7344 × 10^−6^	1.7344 × 10^−6^	1.7344 × 10^−6^	2.5846E-03	1.7344 × 10^−6^
F30	1.7344 × 10^−6^	1.7344 × 10^−6^	1.7344 × 10^−6^	1.7344 × 10^−6^	1.7344 × 10^−6^	1.7344 × 10^−6^
	WEMFO	DE	PSO	SMA	TSA	
F1	2.2248 × 10^−4^	6.2884E-01	1.7344 × 10^−6^	1.7344 × 10^−6^	1.7138 × 10^−1^	
F2	2.5637 × 10^−2^	1.7344 × 10^−6^	1.5286 × 10^−1^	4.9080 × 10^−1^	1.7344 × 10^−6^	
F3	2.6033 × 10^−6^	1.7344 × 10^−6^	6.3391 × 10^−6^	5.7517 × 10^−6^	1.7344 × 10^−6^	
F4	1.6394 × 10^−5^	1.3595 × 10^−4^	8.2167 × 10^−3^	2.8434 × 10^−5^	2.6033 × 10^−6^	
F5	3.5152 × 10^−6^	1.3595 × 10^−4^	1.7344 × 10^−6^	3.7243 × 10^−5^	1.7344 × 10^−6^	
F6	1.7344 × 10^−6^	1.7344 × 10^−6^	1.7344 × 10^−6^	1.7344 × 10^−6^	1.7344 × 10^−6^	
F7	2.3534 × 10^−6^	1.4704 × 10^−1^	1.7344 × 10^−6^	1.7344 × 10^−6^	1.7344 × 10^−6^	
F8	1.7344 × 10^−6^	6.1564 × 10^−4^	1.7344 × 10^−6^	3.5009 × 10^−2^	1.7344 × 10^−6^	
F9	1.7344 × 10^−6^	1.7344 × 10^−6^	1.7344 × 10^−6^	1.7344 × 10^−6^	1.1561 × 10^−1^	
F10	1.3194 × 10^−2^	4.9080 × 10^−1^	9.0993 × 10^−1^	5.2165 × 10^−6^	1.7344 × 10^−6^	
F11	2.1266 × 10^−6^	1.8519 × 10^−2^	1.7344 × 10^−6^	7.1570 × 10^−4^	3.8822 × 10^−6^	
F12	1.9209 × 10^−6^	1.7344 × 10^−6^	1.7344 × 10^−6^	1.7344 × 10^−6^	1.7344 × 10^−6^	
F13	1.7344 × 10^−6^	1.3601 × 10^−5^	1.7344 × 10^−6^	1.1138 × 10^−3^	4.6528 × 10^−1^	
F14	1.7344 × 10^−6^	1.7344 × 10^−6^	9.3157 × 10^−6^	1.7344 × 10^−6^	3.1817 × 10^−6^	
F15	2.3534 × 10^−6^	3.1123 × 10^−5^	1.7344 × 10^−6^	5.7924 × 10^−5^	4.7162 × 10^−2^	
F16	2.8021 × 10^−1^	1.7344 × 10^−6^	2.4308 × 10^−2^	4.2843 × 10^−1^	1.7344 × 10^−6^	
F17	1.9729 × 10^−5^	8.9443 × 10^−4^	2.6033 × 10^−6^	3.8822 × 10^−6^	1.2866 × 10^−3^	
F18	1.7344 × 10^−6^	1.7344 × 10^−6^	3.1817 × 10^−6^	3.8822 × 10^−6^	1.7344 × 10^−6^	
F19	4.4493 × 10^−5^	1.5658 × 10^−2^	1.7344 × 10^−6^	1.2453 × 10^−2^	8.4508 × 10^−1^	
F20	3.1618 × 10^−3^	1.7344 × 10^−6^	1.7344 × 10^−6^	2.7029 × 10^−2^	3.3789 × 10^−3^	
F21	1.7344 × 10^−6^	9.3157 × 10^−6^	1.7344 × 10^−6^	5.3070 × 10^−5^	1.7344 × 10^−6^	
F22	3.1817 × 10^−6^	1.7344 × 10^−6^	1.7344 × 10^−6^	1.7344 × 10^−6^	2.2888 × 10^−1^	
F23	1.9209 × 10^−6^	7.1903 × 10^−2^	1.9209 × 10^−6^	7.7122 × 10^−4^	1.7344 × 10^−6^	
F24	2.6033 × 10^−6^	5.2165 × 10^−6^	1.7344 × 10^−6^	1.9729 × 10^−5^	1.7344 × 10^−6^	
F25	1.9569 × 10^−2^	2.5364 × 10^−1^	2.7653 × 10^−3^	3.0861 × 10^−1^	2.5364 × 10^−1^	
F26	1.9209 × 10^−6^	1.1973 × 10^−3^	4.5336 × 10^−4^	2.2248 × 10^−4^	2.4118 × 10^−4^	
F27	6.1564 × 10^−4^	2.6033 × 10^−6^	1.1748 × 10^−2^	6.5833 × 10^−1^	9.3157 × 10^−6^	
F28	1.7344 × 10^−6^	4.3896 × 10^−3^	1.7344 × 10^−6^	1.7344 × 10^−6^	9.3157 × 10^−6^	
F29	3.8822 × 10^−6^	3.7243 × 10^−5^	2.1266 × 10^−6^	1.5658 × 10^−2^	1.9729 × 10^−5^	
F30	1.9209 × 10^−6^	4.4493 × 10^−5^	1.7344 × 10^−6^	2.1266 × 10^−6^	1.7344 × 10^−6^	

## Data Availability

Data is unavailable due to privacy or ethical restrictions.

## References

[1] Faris H., Ala’M A.-Z., Heidari A.A., Aljarah I., Mafarja M., Hassonah M.A., Fujita H. (2019). An intelligent system for spam detection and identification of the most relevant features based on evolutionary Random Weight Networks. Inf. Fusion.

[2] Zhang W., Liu J., Liu J., Liu Y., Tan S. (2024). A dual distance dominance based evolutionary algorithm with selection-replacement operator for many-objective optimization. Expert Syst. Appl..

[3] Wang Z.-Z., Zhang K., Chen G.-D., Zhang J.-D., Wang W.-D., Wang H.-C., Zhang L.-M., Yan X., Yao J. (2023). Evolutionary-assisted reinforcement learning for reservoir real-time production optimization under uncertainty. Pet. Sci..

[4] Zhuang X., Wang W., Su Y., Yan B., Li Y., Li L., Hao Y. (2024). Multi-objective optimization of reservoir development strategy with hybrid artificial intelligence method. Expert Syst. Appl..

[5] Storn R., Price K. (1997). Differential Evolution—A Simple and Efficient Heuristic for Global Optimization over Continuous Spaces. J. Glob. Optim..

[6] Dorigo M., Birattari M., Stutzle T. (2007). Ant colony optimization. IEEE Comput. Intell. Mag..

[7] Kennedy J., Eberhart R. Particle swarm optimization. Proceedings of the IEEE International Conference on Neural Networks—Conference Proceedings.

[8] Abdel-Basset M., Mohamed R., Azeem S.A.A., Jameel M., Abouhawwash M. (2023). Kepler optimization algorithm: A new metaheuristic algorithm inspired by Kepler’s laws of planetary motion. Knowl. Based Syst..

[9] Dehghani M., Montazeri Z., Trojovská E., Trojovský P. (2023). Coati Optimization Algorithm: A new bio-inspired metaheuristic algorithm for solving optimization problems. Knowl. Based Syst..

[10] Zhao Z., Luo S. (2024). A Crisscross-Strategy-Boosted Water Flow Optimizer for Global Optimization and Oil Reservoir Production. Biomimetics.

[11] Song Y., Cai X., Zhou X., Zhang B., Chen H., Li Y., Deng W., Deng W. (2023). Dynamic hybrid mechanism-based differential evolution algorithm and its application. Expert Syst. Appl..

[12] Li L., Liu L., Shao Y., Zhang X., Chen Y., Guo C., Nian H. (2023). Enhancing Swarm Intelligence for Obstacle Avoidance with Multi-Strategy and Improved Dung Beetle Optimization Algorithm in Mobile Robot Navigation. Electronics.

[13] Bian X., Zhang R., Liu P., Xiang Y., Wang S., Tan X. (2023). Near infrared spectroscopic variable selection by a novel swarm intelligence algorithm for rapid quantification of high order edible blend oil. Spectrochim. Acta Part A Mol. Biomol. Spectrosc..

[14] Chen A., Liao Y., Cai H., Guo X., Zhang B., Lin B., Zhang W., Wei L., Tong Y. (2023). Experimental study on 3D source localization in indoor environments with weak airflow based on two bionic swarm intelligence algorithms. Build. Environ..

[15] Wolpert D.H., Macready W.G. (1997). No free lunch theorems for optimization. IEEE Trans. Evol. Comput..

[16] Kiran M.S. (2015). TSA: Tree-seed algorithm for continuous optimization. Expert Syst. Appl..

[17] Babalik A., Cinar A.C., Kiran M.S. (2018). A modification of tree-seed algorithm using Deb’s rules for constrained optimization. Appl. Soft Comput..

[18] Aslan M., Beskirli M., Kodaz H., Kiran M. (2018). An improved tree seed algorithm for optimization problems. Int. J. Mach. Learn Comput..

[19] Eskandar H., Sadollah A., Bahreininejad A., Hamdi M. (2012). Water cycle algorithm—A novel metaheuristic optimization method for solving constrained engineering optimization problems. Comput. Struct..

[20] Korashy A., Kamel S., Youssef A.-R., Jurado F. (2019). Modified water cycle algorithm for optimal direction overcurrent relays coordination. Appl. Soft Comput..

[21] Osaba E., Ser J.D., Sadollah A., Bilbao M.N., Camacho D. (2018). A discrete water cycle algorithm for solving the symmetric and asymmetric traveling salesman problem. Appl. Soft Comput..

[22] El-Fergany A.A., Hasanien H.M. (2019). Water cycle algorithm for optimal overcurrent relays coordination in electric power systems. Soft Comput..

[23] Elhameed M.A., El-Fergany A.A. (2017). Water cycle algorithm-based economic dispatcher for sequential and simultaneous objectives including practical constraints. Appl. Soft Comput..

[24] Coelho L.d.S. (2010). Gaussian quantum-behaved particle swarm optimization approaches for constrained engineering design problems. Expert Syst. Appl..

[25] Zhu A., Xu C., Li Z., Wu J., Liu Z. (2015). Hybridizing grey wolf optimization with differential evolution for global optimization and test scheduling for 3D stacked SoC. J. Syst. Eng. Electron..

[26] Kumar N., Hussain I., Singh B., Panigrahi B.K. (2017). Single sensor-based MPPT of partially shaded PV system for battery charging by using cauchy and gaussian sine cosine optimization. IEEE Trans. Energy Convers..

[27] Luo J., Chen H., Heidari A.A., Xu Y., Zhang Q., Li C. (2019). Multi-strategy boosted mutative whale-inspired optimization approaches. Appl. Math. Model..

[28] Gupta S., Deep K. (2019). A hybrid self-adaptive sine cosine algorithm with opposition based learning. Expert Syst. Appl..

[29] Nenavath H., Jatoth R.K. (2018). Hybridizing sine cosine algorithm with differential evolution for global optimization and object tracking. Appl. Soft Comput..

[30] Shan W., Qiao Z., Heidari A.A., Chen H., Turabieh H., Teng Y. (2021). Double adaptive weights for stabilization of moth flame optimizer: Balance analysis, engineering cases, and medical diagnosis. Knowl. Based Syst..

[31] Abbassi R., Abbassi A., Heidari A.A., Mirjalili S. (2019). An efficient salp swarm-inspired algorithm for parameters identification of photovoltaic cell models. Energy Convers. Manag..

[32] Zhang Q., Chen H., Heidari A.A., Zhao X., Xu Y., Wang P., Li Y., Li C. (2019). Chaos-induced and Mutation-driven Schemes Boosting Salp Chains-inspired Optimizers. IEEE Access.

